# Dietary Phytoadditives Enhance Growth Performance, Digestive Enzyme Activity, Antioxidant Status, Immune Gene Expression, and *Vibrio parahaemolyticus* Resistance in Pacific White Shrimp (*Penaeus vannamei*)

**DOI:** 10.1155/anu/2575043

**Published:** 2026-10-22

**Authors:** Elangovan Prabu, Nathan Felix, Victor L. Nsereko, Jarin Sawanboonchun, Pushparaj Chidambaram, Arumugam Uma, Thangaraju Thiruvasagam, Anbazhagan Monishkumar

**Affiliations:** ^1^ Tamil Nadu Dr. J. Jayalalithaa Fisheries University Directorate of Incubation and Vocational Training in Aquaculture ECR-Muttukadu Chennai 603112 Tamil Nadu India tnjfu.ac.in; ^2^ USAID-TRANSFORM Cargill Inc. Minneapolis 55440-9300 Minnesota USA; ^3^ USAID-TRANSFORM Cargill Inc. Pathum Wan Bangkok Thailand

**Keywords:** immunostimulants, *Penaeus vannamei*, phytoadditives, tamarind, *Vibrio parahaemolyticus*

## Abstract

The present study evaluated the effects of dietary phytoadditive supplementation on growth, nutrient utilization, physiological responses, and disease resistance against *Vibrio parahaemolyticus* in Pacific white shrimp (*Penaeus vannamei*). Four diets, isonitrogenous, isolipidic, and isocaloric, included phytoadditives (turmeric, garlic, ginger, and tamarind in 0.25:0.25:0.25:0.25 ratio) at 0% (control), 0.5%, 1%, and 1.5%. Juvenile shrimp with an initial mean weight of 1.57 ± 0.03 g were randomly placed into 16 tanks. Each tank contained 500 L of water and 50 shrimp. Each treatment diet was allocated to four replicate tanks (*n* = 4). The shrimp were fed four times daily at 06.00, 10.00, 14.00, and 18.00 H for 60 days. At the end of the feeding trial, dietary supplementation with the combined phytoadditives significantly improved the growth performance and nutrient utilization of *P. vannamei*. Shrimp fed the 1% combined phytoadditives had higher (*p* < 0.05) weight gain (16.14 ± 0.32 g), specific growth rate (4.03 ± 0.06% day^−1^), and protein and lipid efficiency ratios (1.79 ± 0.05 and 12.10 ± 0.37) than those fed the other diets, with a correspondingly lower feed conversion ratio (1.53 ± 0.05). However, the combined phytoadditives did not significantly affect the whole‐body nutrient composition of *P. vannamei*. Shrimp fed the 1% combined phytoadditive diet showed significantly (*p* < 0.05) higher protease (23.02 ± 1.85 U/mg protein % over control) and lipase (18.13 ± 1.52 U/mg protein) activities in the intestine and amylase activity (2.78 ± 0.21 U/mg protein) in the hepatopancreas. Conversely, intestinal amylase, hepatopancreatic protease, and lipase activities were not significantly influenced by the different inclusion levels of the combined phytoadditives. Shrimp fed the 1% combined phytoadditive diet showed significantly higher (*p* < 0.05) superoxide dismutase (SOD) (29.43 ± 2.52 U/mg protein) and glutathione peroxidase (GPx) (0.009 ± 0.001 U/mg protein) activities in the intestine and catalase (CAT) activity (1.30 ± 0.22 U/mg protein) in the hepatopancreas. In contrast, CAT activity in the shrimp intestine and SOD and GPx activities in the hepatopancreas were not significantly affected by the dietary treatments (*p* > 0.05). Significantly upregulated Toll‐like receptor (TLR), immune deficiency (IMD), and lysozyme (LYZ) relative mRNA expression was observed in shrimp fed a diet supplemented with 1% combined phytoadditives. Dietary supplementation with 1% combined phytoadditives significantly reduced cumulative mortality during the challenge study (28.12 ± 6.25% versus 53.12 ± 6.25% in the control), corresponding to a relative percent survival (RPS) of 47.1%, compared with 17.6% and 38.9% at the 0.5% and 1.5% inclusion levels, respectively. In conclusion, dietary supplementation with 1% combined phytoadditives improved overall growth performance and immune response in Pacific white shrimp.

## 1. Introduction

Shrimp farming is considered a significant aquaculture activity because of its delicious taste, high income generation in international markets, and contribution to employment creation [1]. Pacific white shrimp (*Penaeus vannamei*) are extensively farmed because of their rapid growth, delicate texture, and outstanding nutritional benefits among shrimp species [2]. Consequently, *P. vannamei* contributed 6.8 million tons to global aquaculture production in 2022 [3]. However, intensive stocking densities and environmental changes have led to disease outbreaks, resulting in significant economic losses in global shrimp production [4–6]. Shrimp farming has been severely affected by bacterial diseases, with *Vibrio* species being major causative agents of various diseases and substantial economic losses [7]. Among these, acute hepatopancreatic necrosis disease (AHPND) and white feces disease (WFD) are the most devastating, leading to massive mortality [8, 9]. *Vibrio parahaemolyticus* is the sole causative agent of AHPND in shrimp culture [10, 11], whereas WFD is caused by a microsporidian parasite, *Enterocytozoon hepatopenaei* (EHP), often in conjunction with *Vibrio* spp. [12].

Antibiotics are routinely administered in shrimp aquaculture to control and prevent disease outbreaks. However, their accumulation in the environment and animal tissues has led to the emergence of antibiotic‐resistant pathogens and potential risks to consumer health [13]. India is the third largest shrimp‐producing country in the world [14]. In recent years, farmers have experienced a steady decline in the farm gate price of *P. vannamei*, with an average global reduction of 2 USD per kilogram. These declining prices, coupled with production losses from disease outbreaks, have driven farmers to seek alternative natural products to replace expensive probiotics and medicines. These alternatives must be locally available, cost‐effective, and capable of preventing production losses caused by diseases [15]. Phytoadditives are plant‐derived compounds added to animal diets to improve health and growth performance. Herbal products are readily available, cost‐effective, and environmentally friendly, with fewer residues and side effects in animal production [16]. These plants provide benefits to shrimp and fish cultures, including growth promotion, stress reduction, immune stimulation, and antimicrobial activity [17]. Recent work has similarly demonstrated that marine polysaccharide–based and other functional feed additives modulate digestive, antioxidant, and innate immune pathways in penaeid shrimp, supporting their use as sustainable alternatives to antibiotic growth promoters and disease‐control agents [18–20]. These effects are attributed to bioactive compounds, such as phenolics, essential oils, pigments, alkaloids, terpenoids, steroids, and flavonoids [21].

Among the herbal products, turmeric (*Curcuma longa* Linn.) is a root plant from the Zingiberaceae family. It grows in many places, especially in Southeast Asia [22]. The main active part of turmeric is curcumin, along with other bioactive compounds like phenolics, flavonoids, β‐carotene, and vitamins C and E. Turmeric has many health benefits. It can lower blood sugar; fight germs, fungi, and viruses; and reduce inflammation. It also has antioxidant, anticancer, liver‐protecting, and brain‐protecting properties [23]. Garlic (*Allium sativum*) belongs to the Amaryllidaceae family. It is used to add flavor to food and in traditional medicine [15, 24]. Garlic has many health benefits. It acts as an antioxidant, fights germs, protects the liver, aids digestion, reduces inflammation, fights cancer, and helps control cholesterol. These benefits are attributed to its active components, such as alliin, allicin, and ajoene [15].

Ginger (*Zingiber officinale*) is a plant native to Southeast China. It is a member of the Zingiberaceae family. Ginger contains many active compounds, such as gingerol and shogaols. These compounds provide ginger benefits, such as protecting the liver, acting as an antioxidant, and aiding digestion [15]. Tamarind (*Tamarindus indica* L.) is a tree of the Fabaceae family. The fruit pulp is used as a spice in Asia, particularly in southern India [25]. Tamarind pulp contains many active substances, such as flavonoids and essential oils, including carvacrol, lupeol, limonene, benzyl benzoate, tamarindienal, and cinnamaldehyde [26, 27]. Parts of the tamarind plant have antioxidant and antimicrobial properties [25, 28]. These plants are used in aquafeeds to aid growth, digestion, and immune response in aquatic species [15, 29–32].

While the individual growth‐promoting, antioxidant, and immunostimulatory properties of turmeric, garlic, ginger, and tamarind have each been documented in aquaculture species, and recent studies have extended this evidence base to marine polysaccharides and other functional additives [18–20], no study to date has evaluated the combined, graded‐level effects of these four phytoadditives on growth performance, digestive and antioxidant enzyme activity, innate immune gene expression, and pathogen challenge resistance within a single experimental framework in *P. vannamei*. Therefore, the present study aimed to assess the effects of graded levels of combined phytoadditives (turmeric, garlic, ginger, and tamarind) on growth and immune responses of Pacific white shrimp, providing an integrated, multiendpoint evaluation of a novel functional feed strategy against *V. parahaemolyticus* challenge.

## 2. Materials and Methods

### 2.1. Shrimp Husbandry and Experimental Setup

Healthy postlarvae (PL_12_) of *P. vannamei* were sourced and acclimated in a 10‐ton polypropylene tank. During the 4‐week nursery phase, shrimp were fed a commercial diet containing 36% crude protein and 5% crude lipid at a rate of 8% of their biomass, administered four times daily. Following acclimation, 800 healthy shrimp with uniform size (average weight: 1.57 ± 0.03 g) were randomly distributed into 16 indoor 500‐L polypropylene tanks (stocking density: 50 shrimp/tank). Each dietary treatment was assigned to four replicate tanks (*n* = 4). Shrimp were fed daily at 06:00, 10:00, 14:00, and 18:00 h for a duration of 60 days, with 4% feed rate adopted according to biomass throughout the trial. A daily water exchange of 10% was maintained, along with continuous aeration using a 5 HP blower. Key water quality parameters, including temperature, pH, dissolved oxygen, salinity, ammonia‐N, nitrite‐N, nitrate‐N, hardness, and alkalinity, were monitored and maintained within optimal ranges throughout the experimental period.

### 2.2. Experimental Diet

Four experimental diets, each isoproteic, isolipidic, and isocaloric, were formulated and designated as 0 (control), 0.5, 1, and 1.5 phytoadditives. The phytoadditives consisted of a blend of turmeric, garlic, ginger, and tamarind in equal proportions (0.25:0.25:0.25:0.25, w/w). The control diet was formulated without any phytoadditive supplementation. In contrast, the 0.5, 1, and 1.5 phytoadditive diets incorporated phytoadditives at concentrations of 0.5%, 1%, and 1.5% of the diet, respectively. Table 1 provides detailed information on the ingredients and nutritional compositions of the experimental diets. All feed ingredients were finely ground using a hammer mill, passed through a 180‐micron mesh, and thoroughly mixed. The oil sources (fish oil and soy lecithin), along with all additives and water, were subsequently added to the ground ingredients and homogenized for 15 min using an electric blender (Gaocheng ‐ GC‐MT1200, Mogli Labs, India Pvt. Ltd.). Following the achievement of a proper mix, the pliable dough was steamed at 80°C for 16 min. Once cooled, it was pelletized using a tabletop pelletizer with a 1.6 mm die. The resulting pellets were dried in a hot air oven at 60°C for 12 h to achieve a moisture content of ~10%. Subsequently, the dried pellets were sealed in airtight plastic containers and stored at 4°C until further use.

**Table 1:** Ingredient composition and nutritional composition (%) of experimental diets.

Ingredients	Phytoadditives			
	Control—0%	0.5%	1%	1.5%
Fish meal^a^	15	15	15	15
Squid meal^a^	5.2	5.2	5.2	5.2
Soybean meal^b^	33	33	33	33
Corn gluten meal^c^	7.3	7.3	7.3	7.3
Wheat flour^b^	21	21	21	21
Corn flour^b^	12.25	11.75	11.25	10.75
Phytoadditives^d^	0	0.50	1	1.5
Fish oil^a^	2	2	2	2
Soy lecithin^e^	1.5	1.5	1.5	1.5
DL‐methionine^i^	0.2	0.2	0.2	0.2
Vitamin mix^g^	0.5	0.5	0.5	0.5
Mineral mix^h^	0.5	0.5	0.5	0.5
Vitamin C^f^	0.05	0.05	0.05	0.05
Binder^j^	1	1	1	1
Dicalcium phosphate^f^	0.5	0.5	0.5	0.5

Proximate compositions				

Dry matter (%)	90.76	90.75	90.77	90.92
Crude protein (%)	36.59	36.41	36.46	36.69
Crude fat (%)	5.30	5.15	5.41	5.21
Crude fiber (%)	2.33	2.32	2.31	2.31
Ash (%)	8.26	8.05	8.31	8.72

Amino acid composition				

Arginine	1.90	1.94	1.87	1.95
Histidine	0.87	0.89	0.85	0.83
Isoleucine	1.51	1.52	1.49	1.47
Leucine	6.35	6.18	6.40	6.33
Lysine	0.50	0.62	0.51	0.57
Methionine	0.40	0.38	0.39	0.38
Phenylalanine	2.00	1.99	2.00	2.01
Threonine	1.23	1.25	1.21	1.18
Tryptophan	0.29	0.28	0.27	0.22
Valine	1.73	1.74	1.72	1.70
Cystine	0.56	0.55	0.57	0.54
TSAA Met+Cys	1.13	1.13	1.14	1.14
Tyrosine	1.41	1.40	1.41	1.41
Phe + Tyr	3.17	3.15	3.17	3.17
Glutamic	7.05	6.92	7.02	7.12
Aspartic	1.95	2.14	1.83	1.98
Glycine	1.20	1.28	1.18	1.10
Serine	1.67	1.68	1.66	1.64
Alanine	2.60	2.42	2.58	2.79
*Σ* Essential amino acids	14.97	15.01	14.90	14.79
*Σ* Nonessential amino acids	17.68	17.66	17.60	17.58

^a^Bismi Fisheries Pvt. Ltd., Mayiladuthurai, Tamil Nadu, India.

^b^Mahindra Feeds Pvt. Ltd., Namakkal, Tamil Nadu, India.

^c^SPAC Starch Products (India) Pvt. Ltd., Erode, Tamil Nadu, India.

^d^Phytoadditives (combination of turmeric, garlic, ginger, and tamarind, 0.25:0.25:0.25:0.25 w/w).

^e^Otto Chemie Pvt. Ltd., Mumbai, India.

^f^Jain Industrial Chemicals, Chennai, India.

^g^Anicare Pvt. Ltd., Chennai, Tamil Nadu, India. Composition of vitamin premix (quantity/kg): Vit. A‐10,000,000 IU, Vit. B1‐5000 mg, Vit. B2‐5000 mg, Vit. B3‐6000 mg, Vit. B5‐6000 mg, Vit. B6‐6000 mg, Vit. C‐60,000 mg, Vit. D3‐2,000,000 IU, Vit. E‐10,000 IU, and Vit. H‐200 mg.

^h^Anicare Pvt. Ltd., Chennai, Tamil Nadu, India. Composition of mineral premix (quantity/kg): magnesium—2800 mg, iodine—7.4 mg, iron—7400 mg, copper—1200 mg, manganese—11,600 mg, zinc—9800 mg, chlorides cobalt—4 mg, potassium—100 mg, selenium—4 mg, calcium carbonate—27.25%, phosphorous—7.45 mg, sulfur—0.7 mg, sodium—6 mg, Calpan—200 mg, aluminum—1500 mg, and choline chloride—10,000 mg.

^i^Evonik AG (DL‐methionine: MetAMINO–99%).

^j^PEGABIND, Bentoli Agrinutrition India Pvt. Ltd., Chennai, India.

### 2.3. Evaluation of Growth Performance

At the end of the 60‐day feeding trial, shrimp from each replicate tank were counted and weighed to evaluate the growth performance. The parameters were calculated using the following formulas:

Weight gain WG,g=BW1 g−BW0 g,

Average daily growth ADG,g=BW1 g−BW0gDOC,

Survival rate SR,%=NHfNSi×100,

Specific growth rate SGR,% day−1=Ln BW1−Ln BW0DOC×100,

Feed conversion ratio FCR=TFI gWG g,

Protein efficiency ratio PER=WG gPI g,

Lipid efficiency ratio LER=WG g LI g.

BW_1_, final body weight of shrimp; BW_0_, initial body weight of shrimp; DOC, feeding trial days; NH_*f*_, number of shrimps during final harvesting; NS_*i*_, number of shrimps during initial stocking; TFI, total feed intake of shrimp; PI, protein intake of shrimp; LI, lipid intake of shrimp.

### 2.4. Proximate Composition of Experimental Diets and Shrimp

The shrimp whole‐body carcass samples were freeze‐dried, and both shrimp and feed samples were pulverized into a powder. The analytical procedures outlined by the AOAC [33] were employed to determine the moisture, crude protein, crude fat, and ash contents of the shrimp and feed samples. Moisture content was determined by placing the sample in a hot air oven at 105°C for 6 h until a constant weight was achieved. The crude protein content was assessed using the Kjeldahl method, with a conversion factor of *N* × 6.25. While crude fat was extracted utilizing the Soxhlet method (Socsplus ‐ SCS 04 AS DLSTS, Pelican Equipment, Chennai, Tamil Nadu, India). The ash content was calculated based on the weight difference after incinerating the samples in a muffle furnace at 550°C for 6 h. Gross energy was measured using an IKA C 3000 oxygen bomb calorimeter (India Pvt. Ltd., Bengaluru, India). The proximate compositions of the experimental diets and whole‐body carcasses are summarized in Tables 1 and 2, respectively. The amino acid profiles of the experimental diets were analyzed using near‐infrared spectroscopy (NIRS DS2500 L) (Foss India Pvt. Ltd., Pune, India), and the findings are presented in Table 1.

**Table 2:** Digestive and antioxidant enzyme activities (U mg^−1^ protein) in *P. vannamei* fed diets supplemented with different inclusion levels of combined phytoadditives.

	Control—0%	0.5%	1%	1.5%	*p*‐Value
Digestive enzymes					

Shrimp intestine					

Amylase	3.35 ± 0.08	3.50 ± 0.07	3.84 ± 0.05	3.82 ± 0.03	0.654
Protease	19.08 ± 0.98^b^	21.33 ± 1.16^ab^	23.02 ± 1.85^a^	21.38 ± 0.84^ab^	0.007
Lipase	14.94 ± 0.74^b^	16.72 ± 0.91^ab^	18.13 ± 1.52^a^	16.82 ± 0.70^ab^	0.007

Shrimp hepatopancreas					

Amylase	1.92 ± 0.22^c^	2.02 ± 0.17^c^	2.78 ± 0.21^a^	2.40 ± 0.18^b^	<0.001
Protease	15.45 ± 0.66	15.83 ± 0.92	16.44 ± 1.02	16.27 ± 0.56	0.342
Lipase	23.62 ± 0.88	24.75 ± 0.84	25.41 ± 1.65	24.75 ± 1.58	0.317

Antioxidant enzymes					

Shrimp intestine					

CAT	0.83 ± 0.22	1.07 ± 0.29	1.30 ± 0.10	1.08 ± 0.21	0.071
SOD	24.62 ± 0.92^b^	27.57 ± 0.96^ab^	29.43 ± 2.52^a^	27.57 ± 1.45^ab^	0.009
GPx	0.007 ± 0.001^b^	0.008 ± 0.001^b^	0.009 ± 0.001^a^	0.008 ± 0.001^ab^	0.015

Shrimp hepatopancreas					

CAT	0.94 ± 0.24^b^	0.96 ± 0.11^ab^	1.30 ± 0.22^a^	1.19 ± 0.05^ab^	0.031
SOD	20.88 ± 1.38	20.20 ± 0.56	19.49 ± 0.84	20.35 ± 1.22	0.361
GPx	0.007 ± 0.001	0.007 ± 0.001	0.008 ± 0.002	0.008 ± 0.001	0.034

*Note:* The data are expressed as the mean ± standard deviation (SD) from four replicates per treatment (*n* = 4). Values marked with different superscript letters indicate statistically significant differences, as determined by Tukey’s test (*p* < 0.05).

### 2.5. Enzyme Analysis

#### 2.5.1. Sample Collection

At the end of the feeding trial, six shrimp from each replicate group were dissected, and their intestines and hepatopancreas were sampled. These tissues were weighed, homogenized with a chilled 0.25 M sucrose solution, and centrifuged at 4000 rpm at 4°C for 5 min to extract enzymes. The total soluble protein content was measured according to the method of Bradford [34], with absorbance recorded at 595 nm using a UV spectrophotometer against a bovine serum albumin standard curve.

#### 2.5.2. Digestive and Antioxidant Enzymes

Protease, amylase, and lipase activities were evaluated using established methods with specific substrates: casein, starch, and olive oil, respectively [35–37]. Superoxide dismutase (SOD), glutathione peroxidase (GPx), and catalase (CAT) were evaluated using established protocols with specific substrates: nitro blue tetrazolium (NBT) for SOD and hydrogen peroxide (H_2_O_2_) for both GPx and CAT [38–40].

### 2.6. Immune‐Related Gene Expression

Gill tissues were used to extract RNA using the PureZOL RNA isolation reagent (Takara Bio Inc.). The purity and concentration of the extracted RNA were measured using a NanoDrop spectrophotometer at 260 nm. A total of 2 μg RNA was converted into cDNA using a synthesis kit from Thermo Scientific. Gene expression related to immunity was analyzed using a C1000 Touch thermal cycler‐CFX96 Real‐Time PCR system (Bio‐Rad). Primer pairs were designed for *β*‐actin (GenBank Number AF300705; F: 5′‐CGCGACCTCACAGACTACCT‐3′ and R: 5′‐CTCGTAGGACTTCTCCAGCG‐3′), Toll‐like receptors (TLRs) (GenBank Number DQ923424; F: 5′‐TGGTGCTTTCGTCAAACTTC‐3′ and R: 5′‐AACCTGGCCATACACAATGA‐3′), lysozyme (LYZ) (GenBank Number AY170126; F: 5′CGACCTCGATCAGTACATGG‐3′ and R: 5′‐GTAACCCTGGTGACAAGCCT‐3′), and immune deficiency (IMD) (GenBank Number FJ592176; F: 5′‐ATCGAGGAACGAGACAAGGT‐3′ and R: 5′‐CGTACACTCGGTCGACATTC‐3′) based on earlier studies on *P. vannamei* [41]. The analysis adhered to the protocol described by Livak and Schmittgen: an initial denaturation at 95°C for 10 min, followed by 40 cycles of 95°C for 15 s, annealing at 60–62°C for 30 s, and extension at 72°C for 30 s. Relative mRNA expression was quantitatively assessed using the 2^−*Δ* *Δ*Ct^ method, and a dissociation curve analysis was performed [42].

### 2.7. Challenge Study

After the growth trial, a 14‐day challenge study was performed to evaluate the efficacy of phytoadditives in combating *V. parahaemolyticus* infection. This test was performed in 16 80 L experimental troughs, within an indoor facility. Each dietary group was represented by four replicates, and eight shrimp were stocked in each tank. The *V. parahaemolyticus* strain (Strain Code BG/SRLAAH/2019; GenBank Accession Number MW940877.1) was isolated from previously infected shrimp in India. The strain was incubated in tryptic soy broth (TSB) at 150 rpm for 24 h at 30°C. A bacterial culture solution containing *V. parahaemolyticus* (1 × 10^7^ CFU/mL) was introduced into each tank for the challenge test. The shrimp were fed their respective diets at a 4% feed rate at 06:00, 10:00, 14:00, and 18:00 h throughout the test. Mortality was monitored hourly. After 6 h, 80% of the water in each tank was replaced with the appropriate bacterial culture solution, and the number of dead shrimps was recorded. The cumulative mortality of Pacific white shrimp during the challenge study against *V. parahaemolyticus* infection was performed according to the method of Uma et al. [43].

Cumulative mortality %Number of dead shrimp during and end of challenge studyNumber of shrimp used for the challenge study×100.

Relative percent survival (RPS) was calculated according to Costa et al. [44] as RPS (%) = [1−(% mortality in treatment group/% mortality in control group)] × 100.

### 2.8. Statistical Analysis

The Shapiro–Wilk test was used to evaluate the normality of the data, while Levene’s test was employed to check for homogeneity. Percentage data were subjected to arcsine transformation. A one‐way analysis of variance (ANOVA) was conducted employing SPSS 23.0, and Tukey’s test was subsequently applied to assess the significant differences between the dietary treatment groups (*n* = 4). The results are expressed as mean ± standard deviation (SD).

## 3. Results

### 3.1. Growth Performance and Nutrient Utilization

Dietary supplementation with varying inclusion levels of combined phytoadditives optimized the growth and nutrient utilization of shrimp. *P. vannamei* fed diet with 1% phytoadditive inclusion exhibited significantly increased final body weight, WG, ADG, and PER. A significantly higher SGR was observed with 1% phytoadditives, similar to 0.5% and 1.5% (*p* < 0.05). The LER was significantly higher with 1% phytoadditives, comparable to that with 0.5% phytoadditives (*p* < 0.002). Shrimp fed the 1% phytoadditive inclusion diet had a significantly lower FCR than those fed the other experimental diets. Survival rate was not significantly affected by the inclusion of graded levels of phytoadditives (*p* > 0.05). The control diet resulted in poorer growth performance and nutrient utilization. Table 3 details the growth performance, survival rate, and feed utilization across different phytoadditive levels.

**Table 3:** Growth performance of Pacific white shrimp fed diets included with different levels of combined phytoadditives.

Parameters	Phytoadditives				
	Control—0%	0.5%	1%	1.5%	*p*‐Value
BW_0_ (g)	1.56 ± 0.03	1.58 ± 0.04	1.58 ± 0.03	1.57 ± 0.04	0.946
BW_1_ (g)	15.32 ± 0.41^c^	16.55 ± 0.39^b^	17.72 ± 0.31^a^	16.37 ± 0.33^b^	<0.001
WG (g)	13.76 ± 0.41^c^	14.97 ± 0.36^b^	16.14 ± 0.32^a^	14.80 ± 0.37^b^	<0.001
ADG (g)	0.23 ± 0.01^c^	0.25 ± 0.01^b^	0.27 ± 0.01^a^	0.25 ± 0.01^b^	<0.001
SR (%)	95.50 ± 1.91	95.49 ± 1.91	96.50 ± 1.91	95.98 ± 1.53	0.847
SGR (% day^−1^)	3.80 ± 0.05^b^	3.91 ± 0.04^ab^	4.03 ± 0.06^a^	3.91 ± 0.08^ab^	0.002
FCR	1.72 ± 0.02^a^	1.65 ± 0.03^a^	1.53 ± 0.05^b^	1.68 ± 0.06^a^	<0.001
PER	1.59 ± 0.02^b^	1.67 ± 0.03^b^	1.79 ± 0.05^a^	1.63 ± 0.06^b^	<0.001
LER	10.97 ± 0.14^c^	11.79 ± 0.23^ab^	12.10 ± 0.37^a^	11.45 ± 0.42^bc^	0.002

*Note:* The data are expressed as the mean ± standard deviation (SD) from four replicates per treatment (*n* = 4). Values marked with different superscript letters indicate statistically significant differences, as determined by Tukey’s test (*p* < 0.05). Within a row, values not sharing a common superscript letter differ significantly (*p* < 0.05, Tukey’s test); for FCR, superscript “b” denotes the statistically lowest (most favorable) value.

### 3.2. Proximate Composition

The results exhibited that dietary inclusion with different levels of combined phytoadditives did not significantly (*p* > 0.05) affect the whole‐body nutritional content of *P. vannamei* (Table 4).

**Table 4:** Whole‐body nutrient composition Pacific white shrimp fed diets included with graded levels of combined phytoadditives.

Nutrients (%)	Phytoadditives				
	Control—0%	0.5%	1%	1.5%	*p*‐Value
Moisture	72.57 ± 0.15	72.57 ± 0.17	72.50 ± 0.15	72.63 ± 0.24	0.819
Crude protein	17.02 ± 0.12	17.05 ± 0.05	17.13 ± 0.16	17.03 ± 0.21	0.739
Crude lipid	1.73 ± 0.03	1.78 ± 0.04	1.79 ± 0.43	1.75 ± 0.03	0.160
Total ash	3.48 ± 0.06	3.45 ± 0.06	3.44 ± 0.03	3.46 ± 0.06	0.700

*Note:* The data are expressed as the mean ± standard deviation (SD) from four replicates per treatment (*n* = 4).

### 3.3. Digestive Enzyme Activity

Dietary supplementation with varying inclusion levels of combined phytoadditives significantly influenced protease and lipase activities in the shrimp intestine and amylase activity in the shrimp hepatopancreas (*p* < 0.05) (Table 2). Shrimp fed 1% phytoadditive inclusion had significantly higher intestinal protease and lipase activity than those fed other diets (*p* < 0.05). However, this was not much different from shrimp fed with 0.5% or 1.5% supplements. The supplements did not significantly affect amylase activity in the shrimp intestines (*p* > 0.05). However, shrimp fed 1% phytoadditives had significantly higher amylase activity in their hepatopancreas than those fed other diets (*p* < 0.05). The protease and lipase activities in the hepatopancreas were not significantly affected by the different supplement levels of phytoadditives.

### 3.4. Antioxidant Enzyme Activity

In the shrimp intestine, antioxidant enzyme activities were significantly influenced (*P* < 0.05) by the combined phytoadditive inclusion levels, except for CAT (*p* > 0.05) (Table 2). Shrimp fed the 1% combined phytoadditive diet exhibited significantly higher SOD and GPx activities than those fed the control diet (*p* < 0.05). However, in the shrimp hepatopancreas, antioxidant enzyme activities were not significantly affected by the combined phytoadditive inclusion levels (*p* > 0.05), except for CAT (*P* < 0.05) (Table 2). Shrimp fed the 1% combined phytoadditive diet exhibited significantly higher CAT activity than those fed the control diet (*p* < 0.05).

### 3.5. Gene Expression

Graded levels of combined phytoadditive inclusion had a significant impact (*p*  < 0.001) on the expression of LYZ, TLR, and IMD genes (Figure 1). Increasing the dietary supplementation of combined phytoadditives significantly upregulated LYZ and TLR expression, with the highest expression observed in the 1% phytoadditive group and the lowest in the control group (Figure 1a,b). For IMD, expression was significantly upregulated by phytoadditive supplementation relative to the control and was statistically comparable between the 1% and 1.5% inclusion levels (Figure 1c), indicating that IMD upregulation plateaus at inclusion levels ≥1%.

**Figure 1:**
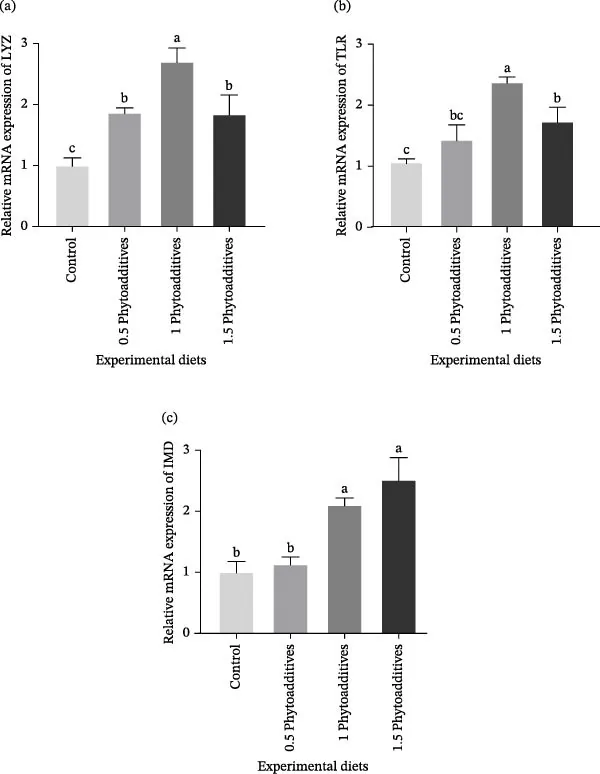
The immune‐related gene expression activities of *P. vannamei* fed graded levels of phytoadditives supplemented diets. (a) Lysozyme. (b) Toll‐like receptors. (c) Immune deficiency. Bars with different superscripts are significantly different (*p* < 0.05).

### 3.6. Challenge Study

Figure 2 shows the results of a 14‐day *V. parahaemolyticus* challenge test. The first mortality occurred 5 h postinfection, with the control group showing 53.12 ± 6.25% mortality. By day 14, groups with 0.5%, 1.5%, and 1% phytoadditives showed lower mortality rates of 43.75 ± 7.22%, 32.43 ± 2.21%, and 28.12 ± 6.25%, respectively, compared to controls. The control diet group had the highest mortality (*p* < 0.05). The corresponding RPS was 17.6%, 47.1%, and 38.9% for shrimp fed 0.5%, 1%, and 1.5% combined phytoadditives, respectively, confirming that the 1% inclusion level conferred the greatest protection against *V. parahaemolyticus* challenge, followed by 1.5% and 0.5% (Figure 3).

**Figure 2:**
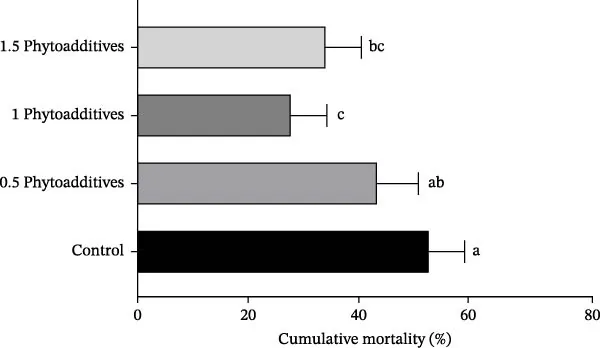
Cumulative shrimp mortality following *V. parahaemolyticus* challenge study. *Note:* Data are presented as mean ± standard deviation (SD) (*n* = 4). Different letters above the bars indicate significant differences among treatment diets (*p* < 0.01).

**Figure 3:**
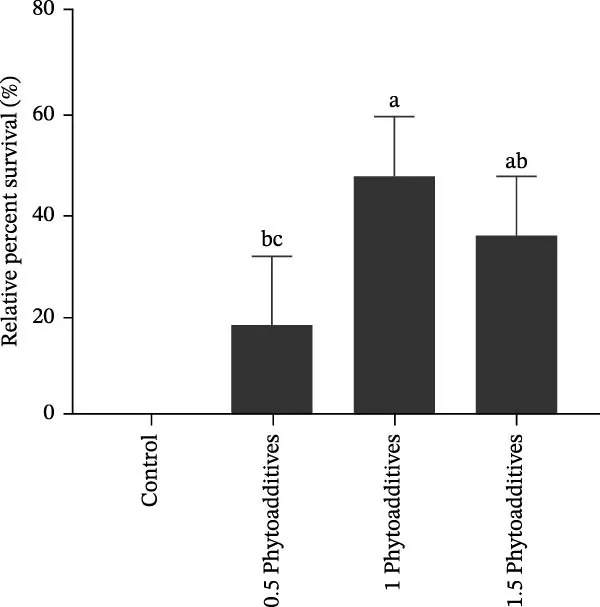
Relative percent survival following *V. parahaemolyticus* challenge study. *Note*: Data are presented as mean ± standard deviation (SD) (*n* = 4). Different letters above the bars indicate significant differences among treatment diets (*p* < 0.01).

## 4. Discussion

Aquatic animals in intensive aquaculture systems are adversely affected by various environmental stressors, such as poor water quality, temperature fluctuations, overcrowding, and pathogen infections. These stressors weaken the immune system, disrupt physiological and metabolic functions, and ultimately lead to reduced growth performance, increased vulnerability to diseases, and substantial production losses [17]. Over the past few years, research has increasingly concentrated on utilizing natural products as feed additives instead of antibiotics to enhance immunity, bolster resistance to environmental stressors and pathogens, and improve the growth performance of aquatic species [15, 30, 31]. This is consistent with recent evidence that functional additives, including marine polysaccharides and phytogenic compounds, enhance disease resistance and immune‐related gene expression in shrimp and other cultured species [18–20].

The present study demonstrated that diets supplemented with graded levels of combined phytoadditives significantly enhanced WG, ADG, and SGR while reducing FCR in *P. vannamei* compared to the control diet. Among the different inclusion levels, shrimp fed a diet with 1% combined phytoadditives showed significantly higher (*p* < 0.05) growth performance and nutrient utilization. Consistent with our findings, previous studies have indicated that dietary supplementation with 6–8 g/kg of garlic improves WGR, SGR, survival, and PER and reduces FCR in *P. vannamei* [24, 30]. Similarly, dietary supplementation with 2.5–5 mg/kg of ginger extract significantly improved weight gain and feed efficiency in *P. vannamei* [45]. García‐Pérez et al. [46] reported that supplementation with 0.2 g/kg of turmeric extract enhanced mean weight, feed intake, and growth rate in *P. vannamei*, even under aflatoxin contamination of feed. To date, no studies have specifically examined the effects of tamarind supplementation on the growth performance and feed efficiency of *P. vannamei*. However, diets fortified with 15–20 g/kg tamarind pulp extract significantly improved growth performance and feed utilization in Nile tilapia (*Oreochromis niloticus*) [28] and African catfish (*Clarias gariepinus*) [29]. The present findings are also consistent with those of studies on narrow‐clawed crayfish (*Pontastacus leptodactylus*) [47], goldfish (*Carassius auratus*) [48], and Nile tilapia [29].

Similar to the current study, Gabriel et al. [49] and Chowdhury et al. [31] found that diets supplemented with combinations of garlic and *Aloe vera* extract (1:1 ratio, at 1.0%) or turmeric and ginger (1:1 ratio, at 1.0%) significantly enhanced the growth performance of African catfish and rohu (*Labeo rohita*), respectively. These positive outcomes can be attributed to the functional properties of phytoadditives, including their antimicrobial activity, stress‐alleviating effects, growth promotion, appetite stimulation, and immune‐enhancing capabilities in aquatic species [17, 50]. Furthermore, the survival rate of shrimp in this study was not significantly affected by supplementation with different levels of combined phytoadditives. This finding confirms that the inclusion of phytoadditives in shrimp diets does not negatively affect survival rates [32, 45, 46, 51].

The whole‐body nutrient composition of aquatic species largely depends on internal factors such as age, sex, and size, as well as external factors such as water quality, season, and geographical area. Nevertheless, diet remains the primary factor influencing chemical composition [52]. The present study revealed that dietary supplementation with different levels of combined phytoadditives had no significant effect on the whole‐body proximate composition, including moisture, crude protein, crude lipid, and ash content, in *P. vannamei*. The lack of significant differences in the whole‐body protein, lipid, fiber, and ash content of Pacific white shrimp across the treatment groups suggests that the phytoadditive‐enriched diets did not substantially alter shrimp nutrient utilization and metabolism. In light of our outcomes, dietary inclusion with garlic‐based diets did not significantly affect the whole‐body crude protein, crude lipid, ash, and moisture content in *P. vannamei* [53]. Dietary supplementation with ginger powder did not significantly affect the whole‐body nutrient composition of olive flounder (*Paralichthys olivaceus*) [54]. However, black garlic supplementation increased the whole‐body nutrient content of Pacific white shrimp [30]. Additionally, dietary supplementation with tamarind pulp extract increased the crude fat and ash content in Nile tilapia, although the crude protein content was unaffected [29]. Chowdhury et al. [31] reported that a diet supplemented with a combination of turmeric, ginger, and garlic decreased the crude fat content but did not affect the crude protein and ash content in the whole‐body proximate composition of rohu. These discrepancies may be attributed to differences in the experimental animal species, their sizes, experimental environmental conditions, inclusion levels, and biochemical compositions of the different phytoadditives used.

Over the last few decades, research has focused on analyzing the digestive enzyme activities of penaeid shrimp to evaluate the digestibility capacity of their diets and the potential of specific ingredients and additives used in their diet. Digestive enzyme activity is directly correlated with diet digestibility and nutrient absorption [55]. In this study, dietary supplementation with different levels of combined phytoadditives significantly enhanced protease and lipase activities in the shrimp intestine, as well as amylase activity in the hepatopancreas. However, the dietary inclusion did not influence amylase activity in the *P. vannamei* intestine or protease and lipase activities in the hepatopancreas. The observed variations in the digestive enzyme activities of shrimp fed diets supplemented with combined phytoadditives can be attributed to the bioactive compounds present in these additives. The significant increase in intestinal protease and lipase activities at 1% supplementation suggests that this is the optimal concentration for enhancing protein and lipid digestion. This improvement may be due to phytoadditives stimulating the secretion of these enzymes or providing cofactors that enhance their activity. The lack of a significant effect on intestinal amylase activity indicates that carbohydrate digestion in the intestine was not substantially influenced by phytoadditives. However, the significant increase in hepatopancreatic amylase activity with 1% supplementation suggests that the phytoadditives may have a tissue‐specific effect on carbohydrate metabolism. The absence of significant changes in hepatopancreatic protease and lipase activities implies that the phytoadditives primarily influence protein and lipid digestion in the intestine rather than in the hepatopancreas. These results collectively indicate that a 1% inclusion level of combined phytoadditives optimizes digestive enzyme activities in *P. vannamei*, potentially leading to improved nutrient utilization and growth performance. Earlier studies have shown that dietary supplementation with phytoadditives can improve gut health by promoting the growth of beneficial microbes, which could potentially modulate the digestive enzyme activities in aquatic animals [56]. Consequently, phytoadditives can enhance the digestibility of aquatic diets [29, 31]. Consistent with our findings, garlic supplementation increased trypsin and amylase activities in the intestines of *P. vannamei* [30]. Dietary supplementation with ginger extract improved protease activity in the shrimp intestine, whereas amylase activity remained unaffected [32]. Ahmed et al. [57] have shown that dietary supplementation with ginger powder and ginger extract stimulated the amylase and protease activities in Nile tilapia. Similarly, dietary supplementation with a combination of turmeric, ginger, and garlic enhanced the protease, amylase, and lipase activities of rohu [31]. Additionally, Adeniyi et al. [29] reported that tamarind leaf extract increased the apparent digestibility coefficient of protein, lipid, and energy in Nile tilapia diets.

Previous studies have reported that supplementation with garlic, ginger, turmeric, and tamarind, which contain bioactive substances with antioxidant properties, effectively alleviates oxidative stress in various aquatic animals [15, 17, 29–31, 58]. In this study, shrimp fed a diet supplemented with 1% combined phytoadditives showed enhanced SOD and GPx activities in the intestine and increased CAT activity in the hepatopancreas. However, CAT activity in the shrimp intestine and SOD and GPx activities in the hepatopancreas were not significantly affected by supplementation with different levels of combined phytoadditives. These results indicate that dietary supplementation with combined phytoadditives significantly influences the antioxidant enzyme activities in *P. vannamei*. The 1% inclusion level of combined phytoadditives showed the most pronounced effects, particularly on intestinal CAT and GPx activities and hepatopancreatic CAT activity. These findings suggest that phytoadditives may enhance the antioxidant defense system in shrimp, potentially improving their ability to cope with oxidative stress. The differential effects on various enzymes and tissues (intestine vs. hepatopancreas) highlight the complex nature of antioxidant responses and suggest tissue‐specific sensitivities to phytoadditive supplementation. The lack of significant effects on some enzymes (e.g., intestinal CAT and hepatopancreatic SOD and GPx) may indicate that these particular antioxidant pathways are less responsive to phytoadditives or that their baseline activities are sufficient to maintain the oxidative balance under the experimental conditions. Similar to our findings, dietary garlic supplementation enhanced total antioxidant capacity (T‐AOC) and increased CAT and SOD activities in *P. vannamei* [30] and Chinese mitten crabs (*Eriocheir sinensis*) [58]. Dietary supplementation with ginger extract (zingerone) improved weight gain and feed efficiency in *P. vannamei*; however, it did not affect SOD activity [45]. In Nile tilapia, the addition of ginger powder and ginger extract improved SOD and CAT activities [57]. Furthermore, Chowdhury et al. [31] demonstrated that a mixture of turmeric, garlic, and ginger elevated CAT activity in the gills of rohu fish. However, SOD activity in the gills and liver, as well as CAT activity in the liver, remained unaffected.

TLR, IMD, and LYZ are crucial genes involved in the immune system of crustaceans. LYZ degrades bacterial cell walls [59, 60]. TLR and IMD genes regulate immune pathways and produce antibacterial peptides [61, 62]. Elevated mRNA levels of these genes indicate a robust immune response in the shrimp. In this study, shrimp fed diets containing 1% and, for IMD, 1.5% phytoadditives exhibited increased mRNA levels of TLR, IMD, and LYZ genes relative to the control. These findings strongly suggest that the combined phytoadditives positively enhanced the innate immune responses in *P. vannamei*, thereby improving growth performance. Similarly, the inclusion of 1 g/kg garlic powder significantly upregulated the TLR, IMD pathway gene (Relish), and LYZ genes in Chinese mitten crabs [58]. Additionally, supplementation of feed with 2 g/kg of turmeric extract enhanced the LYZ expression in the liver of goldfish (*C. auratus*) [48].

Disease outbreaks are a major challenge in shrimp aquaculture. Recently, bacterial diseases such as early mortality syndrome (EMS), also known as WFD and AHPND, have severely impacted shrimp farming. These diseases, caused by the bacterium *V. parahaemolyticus*, result in significant mortality and heavy economic losses for farmers. Earlier studies have shown that the inclusion of phytoadditives as immunostimulants reduces mortality and enhances disease resistance against various bacterial infections in aquatic species [29, 54, 57]. In the present study, after a 14‐day challenge study with *V. parahaemolyticus*, shrimp fed phytoadditive‐supplemented diets showed lower cumulative mortality than those fed the control diet. Shrimp fed 1% combined phytoadditives had significantly lower mortality, likely due to the immunostimulant and antimicrobial properties of turmeric [63], ginger [45], and tamarind [29]. Amoah et al. [30] similarly found that 80 g/kg garlic supplementation reduced mortality due to *V. parahaemolyticus* in Pacific white shrimp. Dietary inclusion with 2.5 and 5 mg/kg of ginger extract (zingerone) improved resistance against *V. alginolyticus* by increasing nonspecific immune parameters, such as phenoloxidase, respiratory burst, phagocytic, and LYZ activities [45]. Additionally, the inclusion of 25 mg/kg turmeric extract enhanced phenoloxidase, phagocytic, and bactericidal activities, leading to reduced cumulative mortality against *Vibrio harveyi* in Pacific white shrimp. Dietary inclusion with 20 g/kg tamarind pulp extract improved the relative survival and reduced the cumulative mortality in Nile tilapia challenged with *Aeromonas hydrophila* infection [28].

The slightly lower protection observed at the 1.5% inclusion level relative to the 1% optimum is consistent with the biphasic, or hormetic, dose–response pattern commonly reported for phytogenic bioactive compounds in aquaculture diets, whereby beneficial immunostimulatory and antioxidant effects at moderate inclusion levels can be attenuated, or even reversed toward mild pro‐oxidant or antinutritional effects, at higher concentrations [17, 46]. Excess intake of curcuminoids, organosulfur compounds (e.g., allicin), and gingerol‐type phenolics has been associated with reduced palatability and feed intake, competitive interactions among coadministered bioactive compounds, and, in some cases, downregulation of antioxidant and immune‐gene pathways once a physiological ceiling is exceeded [16, 17]. The comparatively higher tannin and phenolic load contributed by tamarind pulp at the 1.5% inclusion level may additionally have imposed a mild antinutritional burden, offsetting part of the immunostimulatory benefit observed at the 1% dose. These findings collectively support 1% as the optimal combined‐phytoadditive inclusion level in *P. vannamei* diets rather than a simple dose‐dependent relationship.

## 5. Conclusion

This study demonstrated that dietary supplementation with combined turmeric, garlic, ginger, and tamarind phytoadditives significantly enhanced growth performance, digestive and antioxidant enzyme activities, and immune‐related gene expression and improved resistance to *V. parahaemolyticus* infection in Pacific white shrimp. Shrimp fed the 1% combined‐phytoadditive diet showed the most pronounced improvements, including elevated intestinal protease, lipase, SOD, and GPx activities and hepatopancreatic amylase and CAT activities; significantly upregulated LYZ, TLR, and IMD gene expression; and a reduction in cumulative mortality from 53.12% in control shrimp to 28.12% (RPS = 47.1%) following *V. parahaemolyticus* challenge. These findings extend previous single‐ingredient reports on turmeric, garlic, ginger, and tamarind by demonstrating that their combined, graded‐level inclusion produces a comparable or enhanced multiendpoint response, supporting a synergistic rather than purely additive mode of action. From an applied perspective, a 1% combined‐phytoadditive blend offers shrimp producers a locally available, cost‐effective, antibiotic‐alternative strategy for improving growth and disease resistance, particularly relevant given rising cost pressures and AHPND/WFD‐related losses affecting the global *P. vannamei* industry.

## Funding

This study was funded by the United States Agency for International Development (USAID) (Grant TR‐SUBAG‐HNR‐FAA‐2023‐03).

## Conflicts of Interest

The authors declare no conflicts of interest.

## Data Availability

The data that support the findings of this study are available from the corresponding author upon reasonable request.
